# Probable Rabson-Mendenhall syndrome presenting with acanthosis nigricans: a case report

**DOI:** 10.1093/omcr/omag199

**Published:** 2026-10-25

**Authors:** Mira Akhras, Mohammad Hmaidan, Rahaf Ghazal, Asad Alebrahem, Nemat Alsaghir

**Affiliations:** Department of Dermatology, Faculty of Medicine, Damascus University, Damascus, Syria; Department of Dermatology, Faculty of Medicine, Damascus University, Damascus, Syria; Department of Dermatology, Faculty of Medicine, Damascus University, Damascus, Syria; Department of Pediatrics, Faculty of Medicine, Damascus University, Damascus, Syria; Department of Dermatology, Faculty of Medicine, Damascus University, Damascus, Syria

**Keywords:** acanthosis nigricans, insulin resistance, Rabson Mendenhall syndrome, hyperinsulinemia

## Abstract

Rabson-Mendenhall syndrome (RMS) is a rare autosomal recessive disorder caused by mutations in the insulin receptor gene (INSR), resulting in severe insulin resistance. We report the case of an 8-year-old boy who presented with progressive acanthosis nigricans of the neck, coarse facial features, generalized hypertrichosis, dental abnormalities, a fissured tongue, thickened nails, penile hypertrophy, and hepatomegaly. Laboratory results indicated hyperinsulinemia, severe insulin resistance and fasting hypoglycemia. Based on the clinical and laboratory findings, Rabson–Mendenhall syndrome was considered. Genetic confirmation was not performed because it was unavailable in our country. This case highlights the importance of recognizing acanthosis nigricans as a clinical marker of inherited metabolic syndromes, and the important role of dermatologists in its early recognition.

## Introduction

Rabson-Mendenhall syndrome is a very rare autosomal recessive disorder caused by mutations in the insulin receptor gene, leading to severe insulin resistance [1, 2].

Rabson-Mendenhall syndrome is estimated to affect less than 1 per million people worldwide.

The condition typically shows up in childhood with a mix of distinctive physical signs and metabolic issues. Children often have coarse facial features (such as a broad nose and prominent ears), acanthosis nigricans, generalized hypertrichosis, dental abnormalities like crowded or irregular teeth and a fissured tongue, thickened nails, genital hypertrophy, and sometimes organ enlargement like hepatomegaly [1].

On the metabolic side, there’s extreme hyperinsulinemia, high insulin resistance, fluctuating blood sugars, with fasting hypoglycemia and postprandial hyperglycemia and a risk of early diabetes [2].

## Case report

An 8-year-old boy presented with a complaint of progressively increasing blackish discoloration of the neck (Fig. 1). He was born to consanguineous parents after a full-term uneventful pregnancy. The parents reported that the patient’s brother had similar symptoms, however the brother was not evaluated.

**Figure 1:**
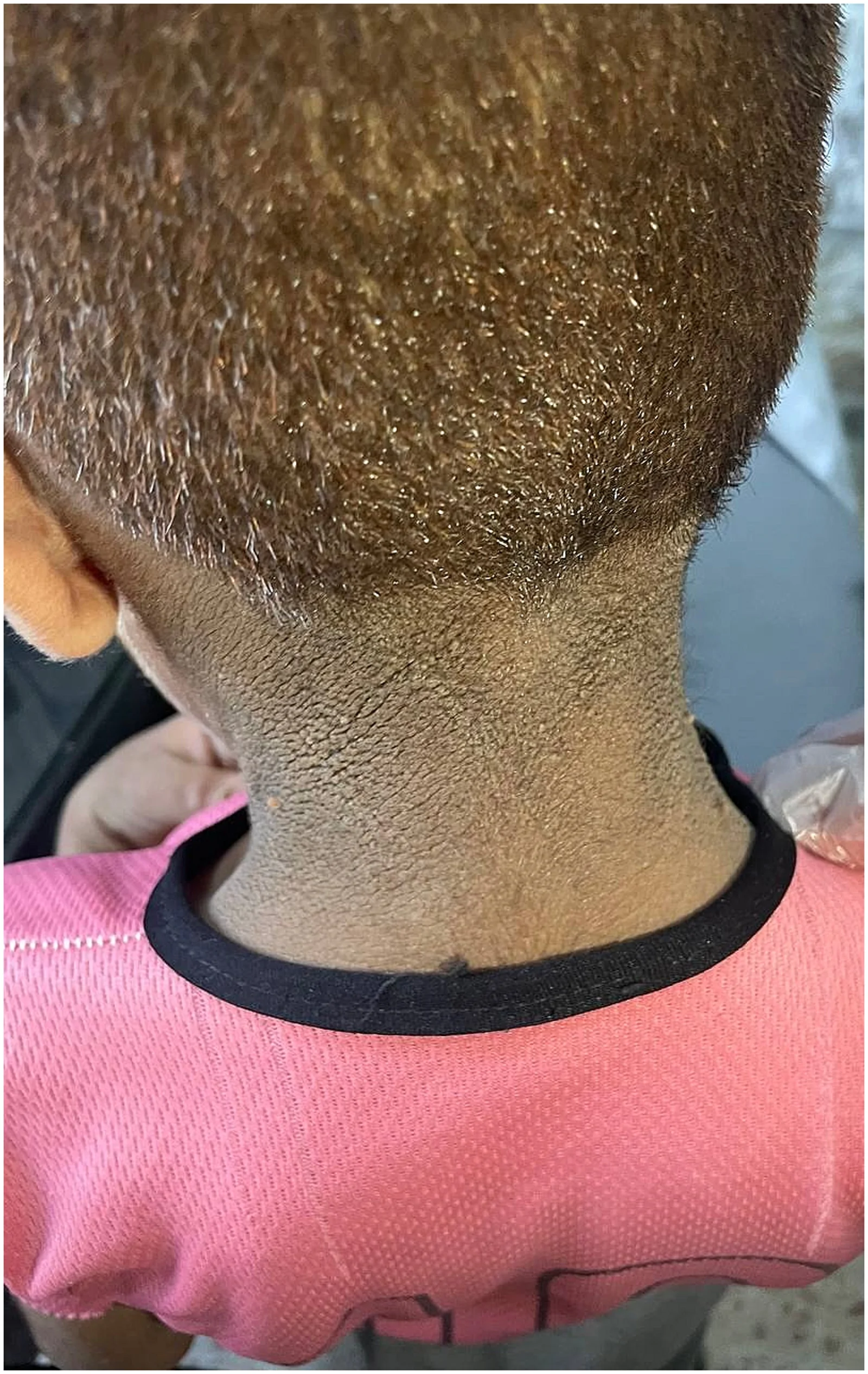
Severe acanthosis nigricans of the neck.

On examination, the boy was found to have coarse facial features including a broad nose and large ears (Fig. 2). Mouth examination showed irregular and crowded teeth and a fissured tongue (Fig. 3 and Fig. 4). Abdominal examination revealed hepatomegaly. Generalized hypertrichosis was also noted.

**Figure 2:**
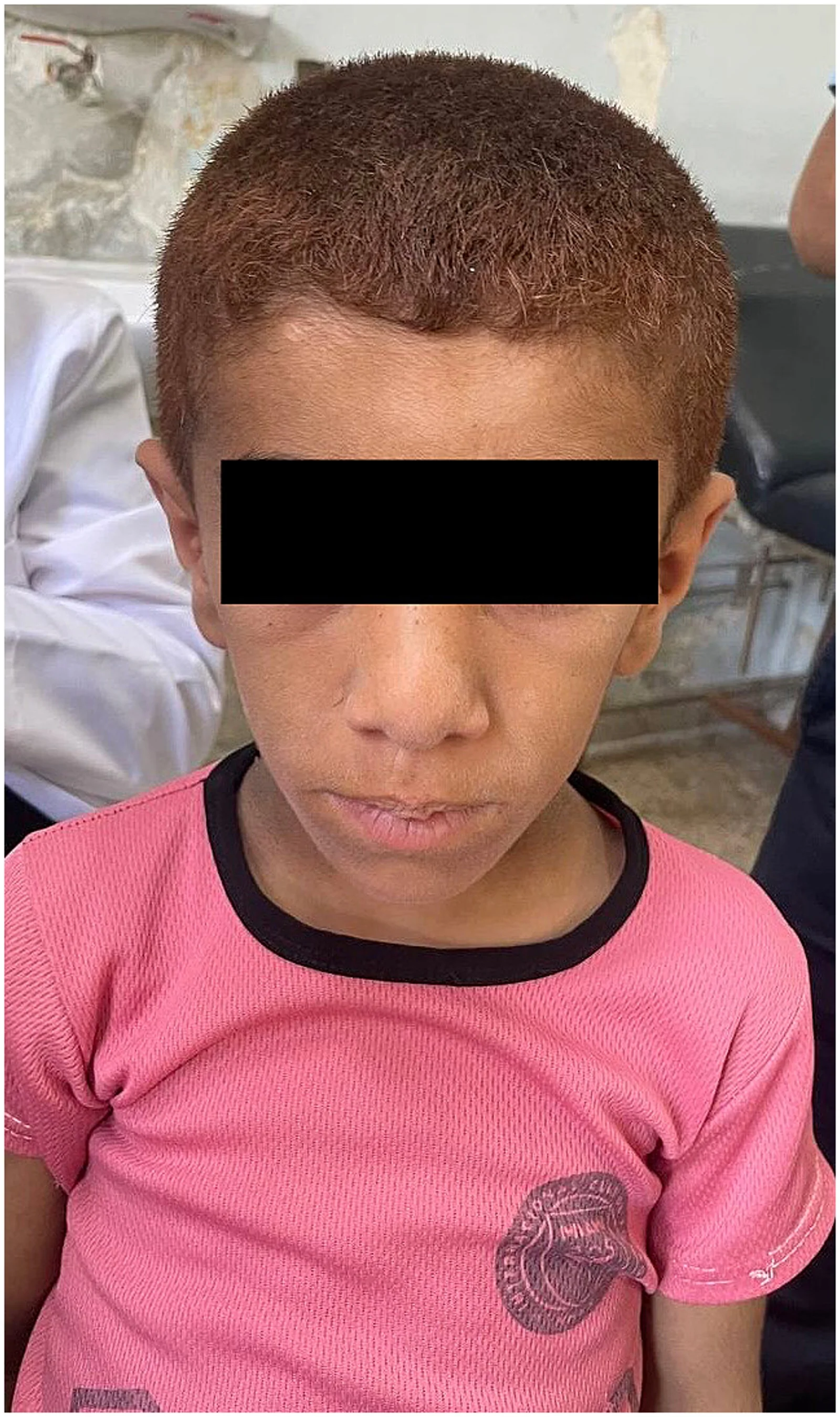
Coarse facial features.

**Figure 3:**
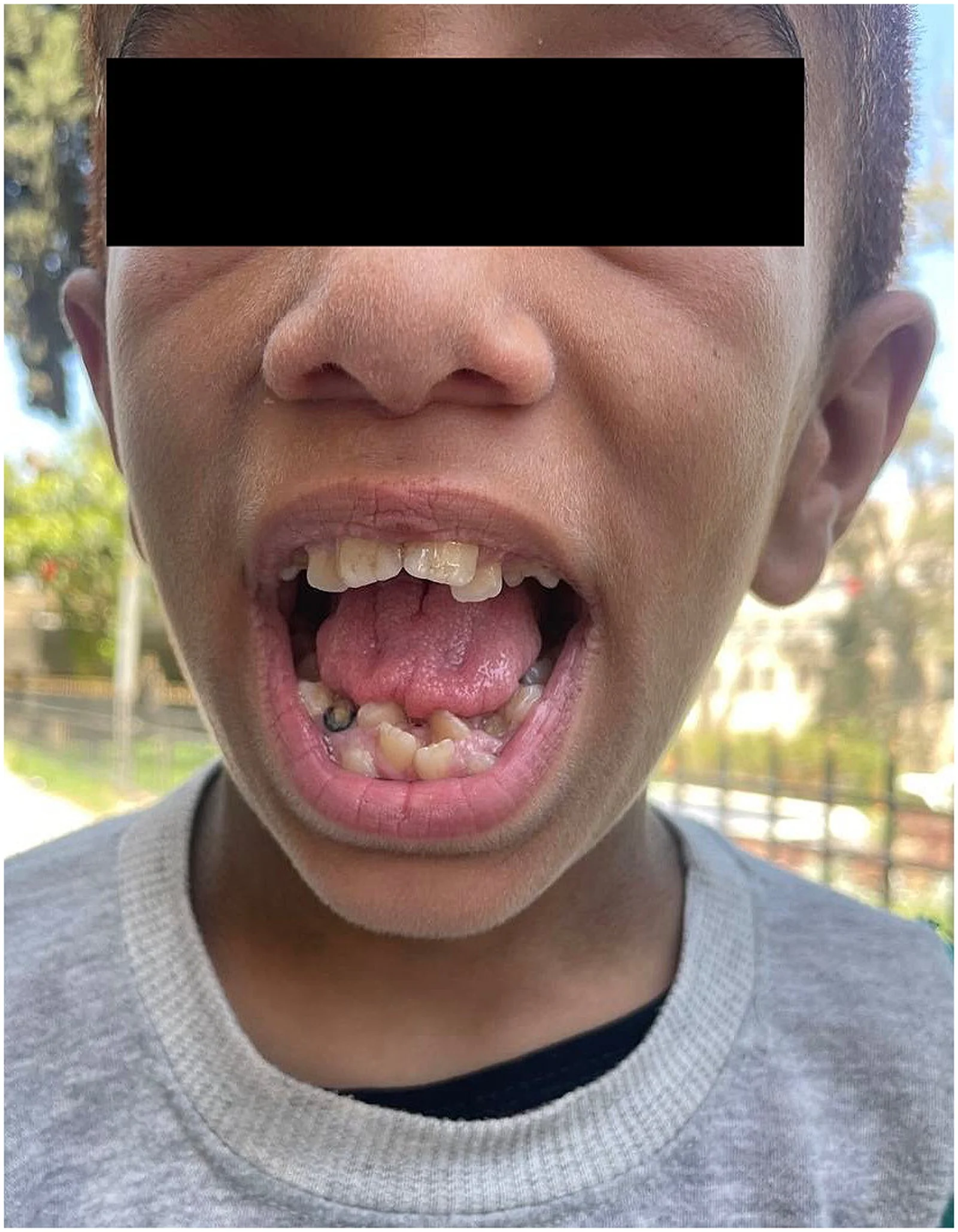
Dental dysplasia, fissured tongue, broad nose, large ears.

**Figure 4:**
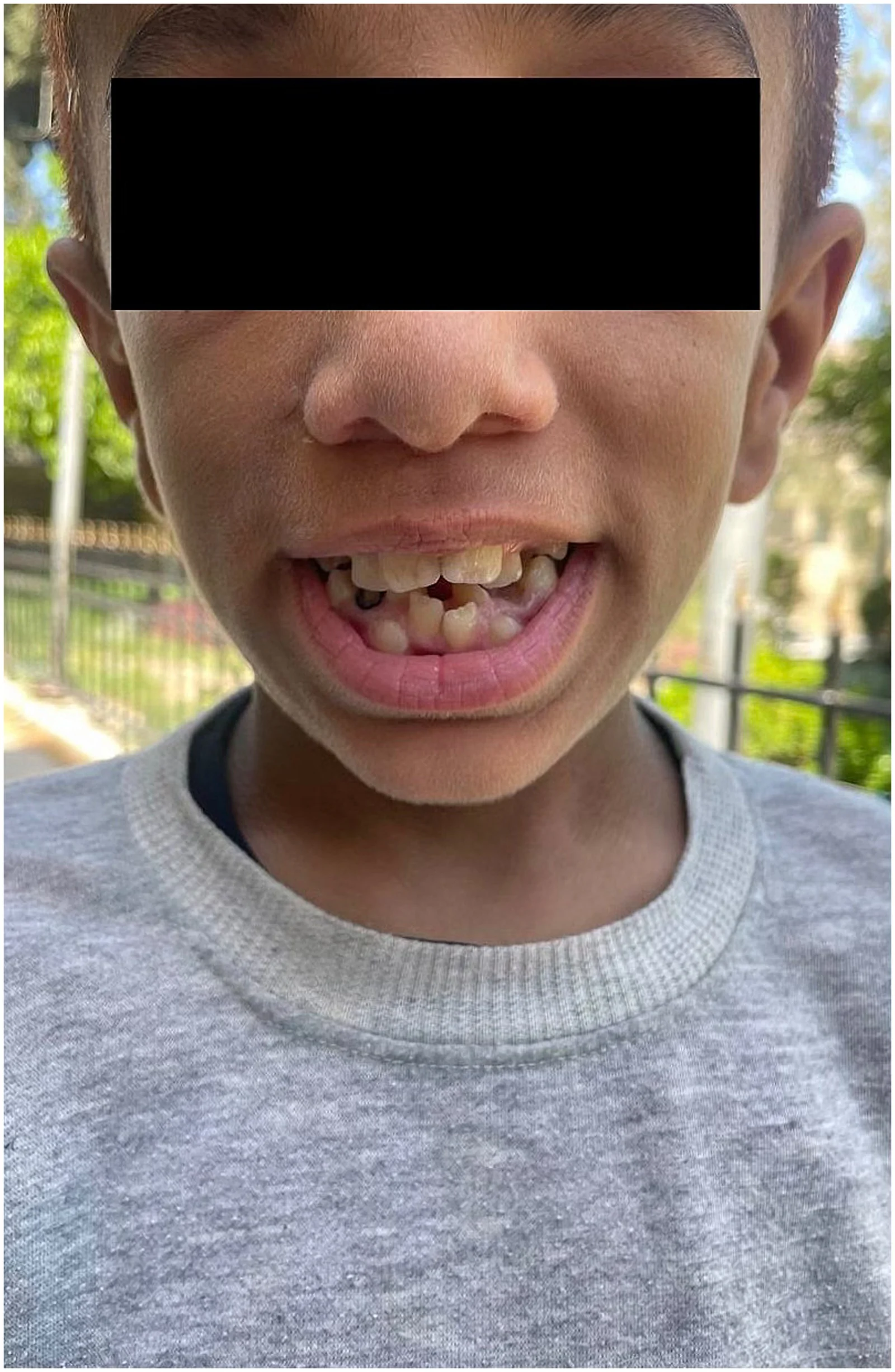
Dental dysplasia, broad nose, large ears.

Extensive acanthosis nigricans was conspicuous on the neck. Examination of the genitals revealed penile hypertrophy. Examination of the nails showed thickened nails (Fig. 5).

**Figure 5:**
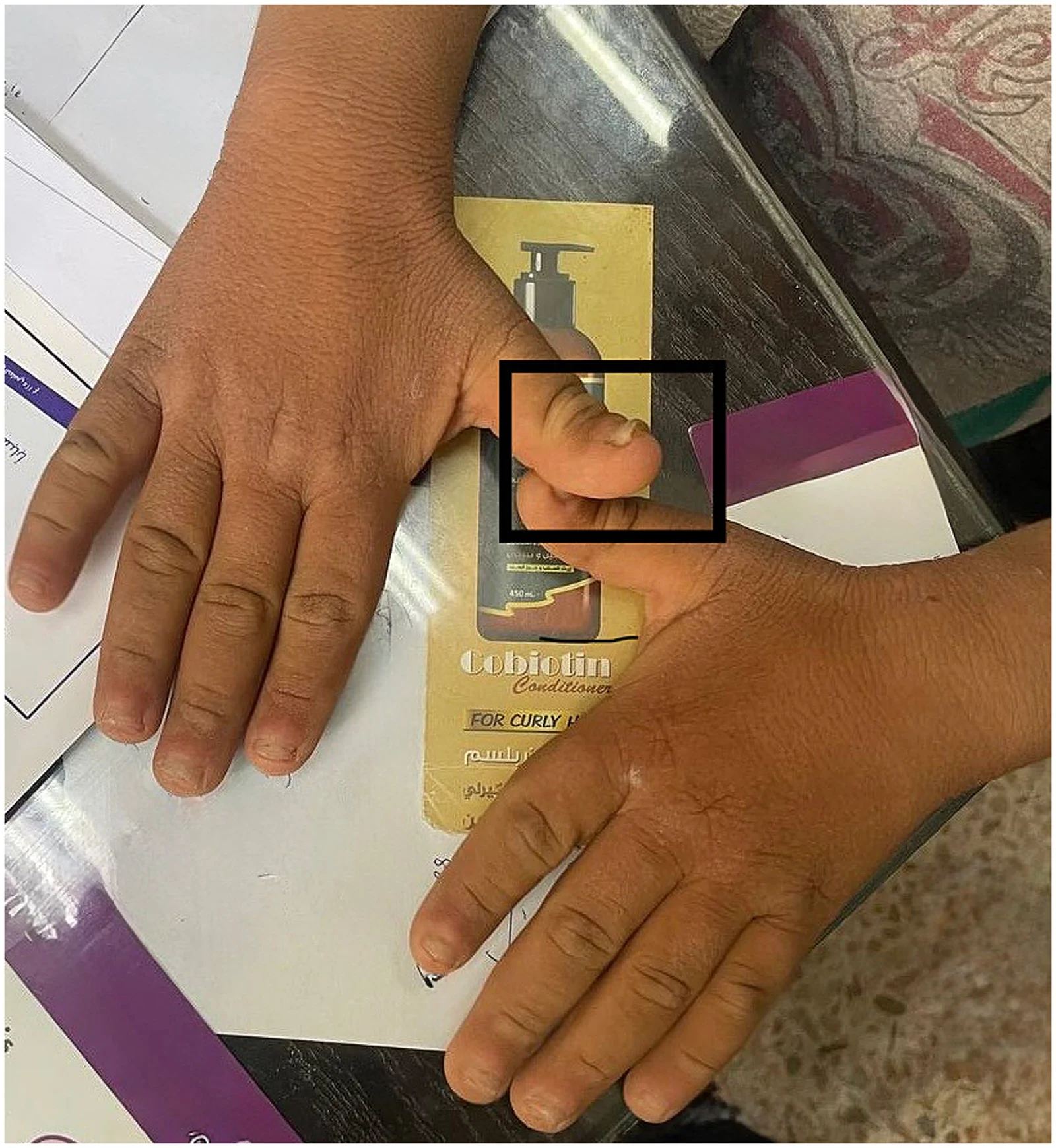
Thickened nails.

Laboratory results revealed elevated serum insulin levels 79 μU/ml, with a HOMA-IR of 10.5. Glycated hemoglobin (HbA1c) was 6.8%,fasting blood sugar was low at 47 mg/dl, serum IGF-1 was 83 ng/ml and serum ketone bodies were 0.7 mmol/l. C-peptide test was not performed.

Routine blood counts, kidney and liver function tests were within normal limits.

Based on the clinical phenotype and laboratory findings, a probable diagnosis of Rabson Mendenhall Syndrome was considered. Genetic confirmation was not performed because genetic testing was unavailable in our country.

Following the diagnosis of probable Rabson-Mendenhall syndrome, the patient was started on topical kligman’s formula to treat the acanthosis nigricans and was referred to a pediatric endocrinologist for further evaluation and management. The patient was lost to dermatological follow-up and the final outcome remains unknown.

## Discussion

Rabson-Mendenhall syndrome (RMS) is a rare inherited insulin resistance syndrome caused by mutations in the INSR gene that lower insulin receptor function, resulting in severe insulin resistance [3].

In the present case, the 8-year-old boy exhibited many of the hallmark clinical features of RMS. These included coarse facial features such as a broad nose and large ears, extensive acanthosis nigricans (most noticeable on the neck), generalized hypertrichosis, dental anomalies with irregular and crowded teeth plus a fissured tongue, thickened nails, penile hypertrophy, and hepatomegaly.

The history of consanguineous parents and the parents’ report of similar symptoms in the patient’s brother align with an inherited disorder. RMS follows an autosomal recessive inheritance pattern. [4, 5]

Laboratory investigations supported the clinical impression of probable RMS, showing markedly elevated fasting insulin (79 μU/ml), a high HOMA-IR index (10.5), mildly elevated HbA1c (6.8%), IGF-1 (83 ng/ml), elevated ketone bodies and fasting hypoglycemia (47 mg/dl), reflecting the severe resistance and hyperinsulinemia seen in RMS. [5]

C-peptide test was not performed because the patient was referred to a pediatric endocrinologist and lost to follow-up.

Genetic testing would have provided confirmation of the diagnosis. However, genetic testing was not available in our country; therefore, it was not performed. And the diagnosis was considered probable based on clinical phenotype and laboratory tests.

RMS overlaps with other genetic insulin resistance syndromes such as Donohue syndrome and type A insulin resistance syndrome. Donohue syndrome represents the most severe phenotype, presenting at birth with intrauterine and postnatal growth restriction, also death usually occurs within the first year of age. While type A insulin resistance presents around puberty with hyperandrogenism and milder metabolic symptoms.

RMS represents a milder phenotype within the spectrum where patients can survive beyond infancy [6].

In the present case the combination of the patient’s age and the coarse facial features favored RMS. In contrast to Donohue syndrome which presents at birth with severe symptoms and type A insulin resistance which presents around puberty with milder symptoms [6], our patient was 8 years old and had RMS features.

Many treatment modalities are now available for children with RMS [7]. High-dose insulin forms the cornerstone, combined with insulin sensitizers such as metformin or pioglitazone [8]. Newer medications, including recombinant IGF-1 and subcutaneous Leptin have shown promising results in some cases [7].

In this case the patient was referred to a pediatric endocrinologist for further evaluation and management. From our side, topical kligman’s formula was prescribed for the treatment of acanthosis nigricans but unfortunately the patient was lost to follow up.

RMS is a very rare syndrome and to the best of our knowledge, this is the first documented case of a probable Rabson-Mendenhall syndrome from Syria.

In addition, the presence of similar symptoms in the patient’s sibling highlights the importance of family evaluation.

As dermatologists, it is very important to consider inherited metabolic syndromes in children presenting with acanthosis nigricans as it may be the initial presenting symptom, and this case underscores the need to recognize the constellation of dysmorphic and metabolic signs, especially in consanguineous families, to facilitate early diagnosis and management of probable RMS.
